# Dianthin and Its Potential in Targeted Tumor Therapies

**DOI:** 10.3390/toxins11100592

**Published:** 2019-10-11

**Authors:** Hendrik Fuchs

**Affiliations:** Charité – Universitätsmedizin Berlin, corporate member of Freie Universität Berlin, Humboldt-Universität zu Berlin, and Berlin Institute of Health; Institute of Laboratory Medicine, Clinical Chemistry and Pathobiochemistry, Augustenburger Platz 1, 13353 Berlin, Germany; hendrik.fuchs@charite.de; Tel.: +49-30-450-569173

**Keywords:** dianthin, ribosome-inactivating protein, targeted toxin, immunotoxin, endosomal escape, cancer therapy, *Dianthus caryophyllus* L.

## Abstract

Dianthin enzymes belong to ribosome-inactivating proteins (RIPs) of type 1, i.e., they only consist of a catalytic domain and do not have a cell binding moiety. Dianthin-30 is very similar to saporin-S3 and saporin-S6, two RIPs often used to design targeted toxins for tumor therapy and already tested in some clinical trials. Nevertheless, dianthin enzymes also exhibit differences to saporin with regard to structure, efficacy, toxicity, immunogenicity and production by heterologous expression. Some of the distinctions might make dianthin more suitable for targeted tumor therapies than other RIPs. The present review provides an overview of the history of dianthin discovery and illuminates its structure, function and role in targeted toxins. It further discusses the option to increase the efficacy of dianthin by endosomal escape enhancers.

## 1. Introduction

Dianthin is the name for three homologous toxins obtained from the clove pink (*Dianthus caryophyllus* L.) and sweet William (*Dianthus barbatus* L.). It was first described by Stirpe and colleagues [1] who isolated two proteins, dianthin-30 and dianthin-32, from the leaves of *D. caryophyllus* L. Another protein, dianthin-29, was isolated by Prestle et al. from frozen leaf material of *D. barbatus* L. [2]. The dianthin enzymes must not be confused with cyclic penta-, hexa-, and hepta-peptides isolated from fringed pink (*Dianthus superbus* L.) and rainbow pink (*Dianthus chinensis* L., *Dianthus sinensis* Link) designated as dianthin A and B [3], dianthin C, D, E, and F [4], dianthin G and H [5,6] and dianthin I [7,8]. These cyclic peptides are not part of this review.

The dianthin enzymes are ribosome-inactivating proteins (RIPs), which are *N*-glycosidases (EC 3.2.2.22) that release a particular adenine from the 28S ribosomal RNA of eukaryotic ribosomes (at position 4324 referred to rat) and thus inactivate protein biosynthesis [9,10]. The concentration of dianthin-30 and dianthin-32 that results in 50% inhibition (IC_50_) of the protein synthesis in a lysate of rabbit reticulocytes was initially determined to be 0.31 nM and 0.11 nM, respectively [1]. The IC_50_ of dianthin-29 in a rabbit reticulocyte lysate was 1.5 nM [2]. Dianthin-29 was described to inhibit in addition prokaryotic ribosomes. Further RIP genes were identified in *D. chinensis* L., exhibiting 79% homology with the dianthin-30 gene [11]. Expression of the proteins in *Escherichia coli* failed, indicating that the translational products of *D. chinensis* L. RIP genes also display toxic effects on prokaryotic ribosomes [11,12]. In contrast, dianthin-30 can be produced in *E. coli* with high yield [13,14], and other RIPs similar to saporin-S6 from *Saponaria officinalis* L. and dianthin-30 were also inactive on bacterial ribosomes [15].

Most of the known RIPs are produced by plants and can be mainly divided into two groups, type 1 RIPs that consist of a single A-chain representing catalytic activity, and type 2 RIPs that, in addition, contain a B-chain with cell binding properties, as compiled by Schrot et al. [16] (Table 1). The fact that type 1 RIPs reveal high cytotoxicity once inside the cell but only low cytotoxicity when located outside the cell due to the missing cell binding domain makes them ideal candidates for targeted tumor therapies as the toxin can be recombinantly fused or chemically coupled to tumor-specific ligands or antibodies, which then mediate cellular uptake [17,18]. Therefore, type 1 RIPs such as saporin and bouganin from *Bougainvillea spectabilis* Willd. have been investigated in a number of attempts as part of targeted toxins in cancer therapy [19,20]. The present review provides an overview of the structure and function of the type 1 RIP dianthin and its potential as an attractive weapon in the fight against cancer.

## 2. Structure and Function

### 2.1. Purification and Expression of Dianthins

Dianthin-30 and dianthin-32 were first purified to homogeneity from the leaves of *D. caryophyllus* L. by chromatography on carboxymethyl (CM-)cellulose, pH 6.5 [1]. The apparent molecular masses determined by sodium dodecyl sulfate polyacrylamide gel electrophoresis (SDS-PAGE) are 29,500 and 31,700 Da respectively, and origin for the naming [1]. Isoelectric focusing provided a single band with a basic isoelectric point of 8.65 for dianthin-30 and 8.55 for dianthin-32, consistent with their chromatographic behavior on CM-cellulose [23]. As determined by rocket immunoelectrophoresis and by the ability to inhibit protein synthesis, dianthin-30 is present throughout the entire plant while dianthin-32 is located only in leaves and growing shoots [24]. In the older parts of the plant, dianthin contributes to 1% to 3% of the total extractable protein, whereas much less is present in the younger parts [24].

The inhibitory activity of dianthin-30 and dianthin-32 was unchanged on pre-incubation at 37 °C for 1 h in the presence of 1% 2-mercaptoethanol, or after freezing and thawing ten consecutive times, or after keeping at 37 °C for 18 h, but was completely abolished by boiling for 20 min [1]. Freeze-dried dianthin-32 retained full activity after solubilization while freeze-dried dianthin-30 was poorly soluble and could not be tested [1].

Dianthin-29 was isolated and purified from frozen leaf material by affinity chromatography on Blue 2 S-Sepharose and subsequent cation exchange chromatography on Mono S [2]. The apparent molecular mass in SDS-PAGE is 29,000 Da [2].

Antibodies are helpful tools for the purification and detection of proteins. Strocchi et al. produced a polyclonal rabbit antiserum against dianthin-32, which showed no cross-reactivity to lychnin from *Lychnis chalcedonica* L. seeds, pokeweed antiviral protein from *Phytolacca americana* L. roots (PAP-R), trichokirin from *Trichosanthes kirilowii* Maxim. seeds, and colocin-1 from *Citrullus colocynthis* (L.) Schrad. seeds, week cross-reactivity to saporin-S6 from *S. officinalis* L. seeds, momordin from *Momordica charantia* L. seeds and momorcochin-S from *Momordica cochinchinensis* (Lour.) Spreng. seeds, and high cross-reactivity to bryodin-R from *Bryonia dioica* Jacq. roots [25]. This antiserum also showed week or medium cross-reactivity with two RIPs isolated from leaves of *Phytolacca dioica* L. [26] and RIPs isolated from the seeds of *Saponaria ocymoides* L., *Vaccaria hispanica* (Mill.) Rauschert [27] and *Basella rubra* L. [28], and from the skinned fruit of *Cucurbita moschata* Duchesne [29]. A rabbit immune antiserum raised against saporin-S6 did not cross-react with dianthin-32 [30]. Porro and colleagues produced five different highly specific anti-dianthin-32 monoclonal antibodies that revealed no cross-reactivity to momochin from *M. cochinchinensis* (Lour.) Spreng. and momordin, and two of them also exhibited no cross-reactivity to saporin-S6, and gelonin from *Gelonium multiflorum* A. Juss. However, all of them bound to dianthin-30, three of them even better than to dianthin-32 [31]. The affinity constants of these anti-dianthin monoclonal antibodies ranged between 1.7 × 10^10^ M^–1^ and 3.3 × 10^8^ M^–1^ [31]. This shows that antibodies against dianthin often exhibit cross-reactivity to RIPs from related plants and sometimes also to RIPs from unrelated plants, as observed for bryodin. Interestingly, four serum samples collected from 17 persons who were employees in a research laboratory and were in daily or in rare contact with RIPs contained specific immunoglobulin E (IgE) against dianthin-30, gelonin, momordin, pokeweed antiviral protein from seeds (PAP-S), saporin, ricin from *Ricinus communis* L. seeds and volkensin from *Adenia volkensii* Harms roots. In contrast, asparin from *Asparagus officinalis* L. seeds and lychnin did not show any IgE binding. Strikingly, among the other plant proteins, RIPs were exclusively recognized by IgE in immunoblots [32].

An important alternative to purification from natural sources is heterologous expression, however, as RIPs inhibit eukaryotic ribosomes, expression in eukaryotic cells is excluded and even expression in prokaryotes is sometimes not possible [11]. Nevertheless, dianthin-30 can be expressed in bacterial systems. This was first described by Legname and colleagues who cloned the cDNA encoding for dianthin-30 into the expression plasmid pKK 233.2 for protein production in *E. coli* strain JM109 [33]. The low amount of 20 µg/L recombinant protein was attributed to the inhibitory effect of dianthin-30 on bacterial protein synthesis as it was shown before that the 23S rRNA of *E. coli* is also a target for dianthin [34]. The yield was substantially increased to 10 mg/L by use of the pET11d expression vector [14]. The authors expressed dianthin-30 and a deleted form (Δ255–270), lacking the putative 16 amino acids long pro-signal sequence, and determined the IC_50_ after purification via cation exchange chromatography on Mono S to 0.29 nM, 0.37 nM, and 0.61 nM for dianthin-30 Δ255–270, recombinant dianthin-30 and natural dianthin-30 respectively, measuring the inhibition of (^14^C)-leucine incorporation into protein using a rabbit reticulocyte system [14]. Investigations by circular dichroism spectroscopy indicated that the natural and the recombinant forms of dianthin-30 possess the same secondary structure composition, accounting for an α + β type architecture [14]. A hexahistidine-tagged dianthin-30 was expressed by Gilabert-Oriol and co-workers in pET11d using *E. coli* Rosetta 2(DE3) pLysS and purified by Ni-nitrilotriacetic acid affinity chromatography eluting at 125 mM imidazole [13]. Mass spectrometry showed a single peak of 29,531 Da, indicating that the *N*-terminal methionine is cleaved off in a bacterial post-translational modification [13]. Circular dichroism measurements conducted after different storage times at −20 °C, 4 °C and 25 °C indicated that the protein stability was not affected by the storage and the protein conformation remained the same. The protein was stable up to 60 °C, where alterations of the structure commenced and achieved 50% denaturation at 66 °C and complete denaturation above 72 °C [13].

### 2.2. Primary and Spatial Structure of Dianthin-30 and Its Catalytic Center

**Figure 1:**
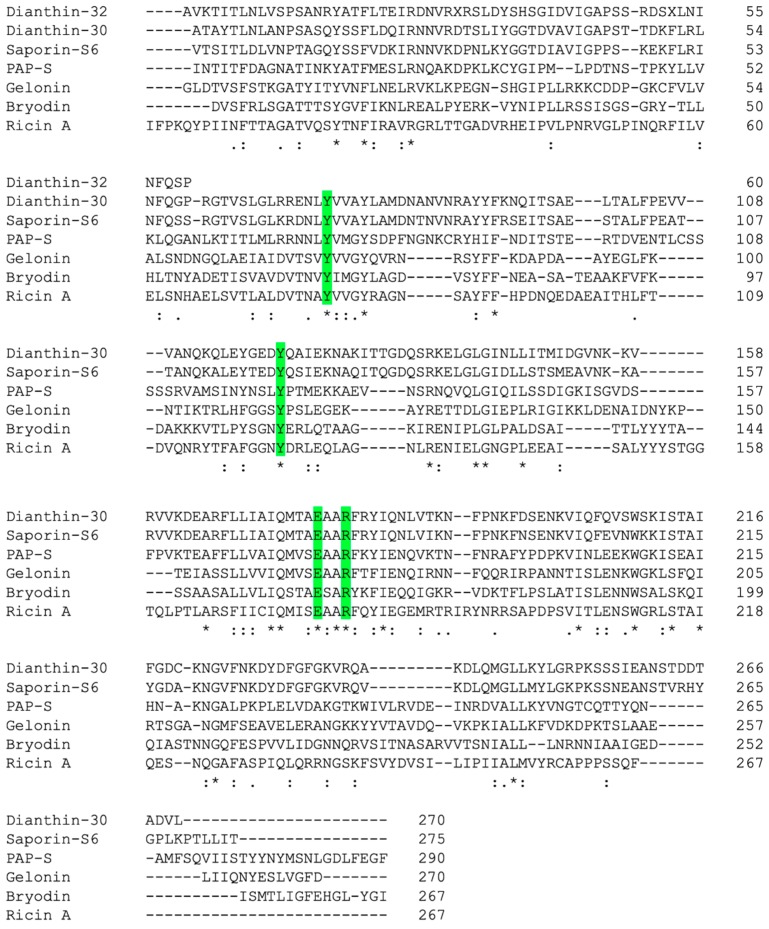
Sequence alignment of the amino acids from dianthin-30 and dianthin-32 (as far as is known) with other RIPs. Asterisks indicate fully conserved residues, colons show strongly similar and periods show weakly similar residues. Highlighted amino acids are involved in the stabilization of the substrate and in the catalysis [38]. Sequences were obtained from the UniProt Knowledgebase (UniProtKB), IDs P24477, P24476, P20656, P93444, P33186, P33185, and P02879. The alignment was conducted with the Clustal Omega tool [39].

Rabbit antibodies raised against dianthin-30 were used to identify a full-length dianthin precursor cDNA clone from a lambda gt11 expression library [35]. The cDNA was 1153 base pairs in length and encoded a precursor protein of 293 amino acid residues, of which the first 23 *N*-terminal amino acids represented the signal sequence [35]. A comparison of the amino acid sequence of dianthin-30 revealed 83% homology with gypsophilin-S from the seeds of *Gypsophila elegans* M. Bieb. [36], 80% homology with saporin-S6 [21] but only 17% with trichosanthin from *T. kirilowii* Maxim. and 20% with ricin A-chain [35] (Figure 1). The *C*-terminal region contains an *N*-glycosylation site and shows homology to a *C-*terminal propeptide present in several plant vacuolar proteins, such as wheat germ agglutinin and barley lectin [35], which is necessary for proper sorting of the lectin to vacuoles [37]. The major sugars observed at the glycosylation site are mannose, glucose and glucosamine for dianthin-32, and xylose and glucose for dianthin-30 [23].

**Figure 2:**
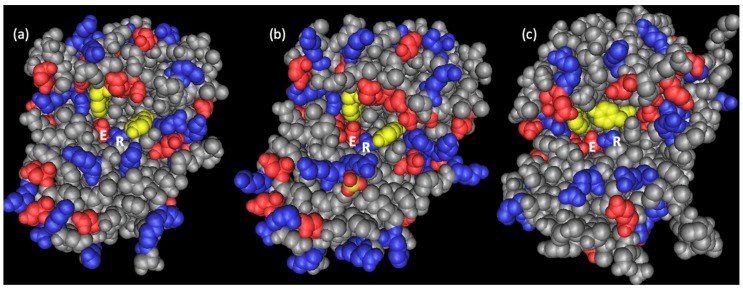
Three-dimensional structure of (**a**) dianthin-30 (Protein Data Bank (PDB) ID: 1RL0) [38], (**b**) saporin-S6 (PDB ID: 1QI7) [47], and (**c**) ricin A-chain (PDB ID: 1RTC) [48]. The yellow residues are the conserved tyrosines in the active center highlighted in Figure 1, the right ones of them corresponding to Tyr-73 of dianthin-30. Negative charged residues are depicted in red and positive residues in blue. The glutamate and arginine of the catalytic pocket are designated with E and R, respectively. The active site of dianthin-30 and saporin-S6 is easily accessible compared to ricin and other RIPs, and saporin-S6 is characterized by a more negative potential compared to dianthin-30 [38].

The three-dimensional structure of dianthin-30 was first predicted by a computer model applying homology modeling on the basis of two known RIP structures from PAP and ricin A-chain [40]. The results demonstrate that, despite the similarity of the topology of the binding site, differences in the electrostatic potential can account for experimentally observed differences in substrate recognition and binding for the investigated RIPs [40]. Experimentally, the structure was independently solved by Kurinov and colleagues for recombinantly expressed dianthin-30 (Δ255–270) (Protein Data Bank (PDB) IDs: 1LP8, 1LPC, 1LPD including complexes with adenyl-guanosine and cyclic AMP) at 1.7 Å resolution [41], and by Fermani et al. for dianthin-30 purified from leaves of *D. caryophyllus* L. (PDB ID: 1RL0) at 1.4 Å resolution [38,42]. Despite some varieties in the loop regions, the typical folding for RIPs is conserved. The structures of dianthin-30 and saporin-S6 fit quite well and both show a protein segment containing strands β7, β8 and β9 that is shorter than in other RIPs, however, the surface electrostatic potential in the active site region distinguishes dianthin-30 from saporin-S6 [38] (Figure 2). While the active site of saporin-S6 is characterized by a negative potential, dianthin-30 contains both a negative potential and an extended positive region in the catalytic cavity [38] corroborating the conclusions from the computer model on differences in the electrostatic potential for PAP and ricin A-chain [40]. The four key residues in the catalytic center, Tyr-73, Tyr-121, Glu-177 and Arg-180, are fully conserved in dianthin-30, saporin-S6, PAP, momordin, trichosanthin, lychnin, bouganin, gelonin, bryodin and ricin A-chain [38,43,44]. Molecular modeling studies of the interactions of dianthin-30 with a single-stranded RNA heptamer predicted a potent anti-human immunodeficiency virus (HIV-) RNA activity due to the unique surface topology and charge distribution in its 20 Å RNA binding cleft. The estimated release was 352 ± 27 pmol adenine per microgram of RNA per hour [41]. Anti-HIV effects were observed for both dianthin-30 and dianthin-32 [45,46].

### 2.3. Enzymatic Activity and Biological Function

All RIPs mediate the release of adenine-4324 (number referred to rat) from the 28S rRNA of eukaryotic ribosomes [9,10]. For dianthin-30 and dianthin-32, this was indirectly confirmed by Reisbig and Bruland who demonstrated that ribosomes remain active when dianthin-treated 40S subunits are combined with untreated 60S subunits but become inactive when treated 60S subunits are combined with untreated 40S subunits [24]. The release of adenine-4324 results in an aldehyde radical at C1 of the ribose [9] and this aldehyde group inhibits the activities of the eukaryotic elongation factor (eEF1A)-dependent aminoacyl-tRNA binding to the inactivated ribosome and eEF1A-dependent guanosine-5’-triphosphatase (GTPase), but increases eEF2-dependent activity [49]. The catalytic activity of RIPs is mainly determined indirectly by in vitro translation assays using rabbit reticulocyte lysate. Similar IC_50_ values between 0.133 nM and 0.61 nM were determined for dianthin-30 in different studies (Table 2). Dianthin-30 and dianthin-32 also inhibited protein synthesis in wheat germ extracts at 0.35–0.70 nM [24], while for ricin A-chain, a similar effect requires more than 800 nM [50]. Moreover, ribosomes from several species are sensitive to their own RIPs, including dianthin-32 [2], saporin [51], and PAP [52]. Notably, the enzymatic activity must not be confused with cytotoxicity as complete ricin possesses a cell binding domain (B-chain) while dianthin can enter cells only by chance. The enzymatic activity of dianthin-30 (Δ255–270), which lacks the glycosylation site, is comparable to that of full-length dianthin-30, indicating that the C-terminus and the sugar residues are not involved in the *N*-glycosidase activity [14]. For saporin-S3 that shares (as saporin-S6) 80% homology with dianthin-30 [21], a complete loss of in vivo activity is observed in the double mutant E176K, R179Q (KQ-mutant) [53], two residues of the catalytic center (residues 177 and 180 in dianthin-30) [38]. Therefore, it can be expected that in dianthin-30, the analogous KQ-mutant will also result in enzymatic activity loss.

As mentioned before, in several cases, RIPs can also inhibit prokaryotic ribosomes. Ferreras and colleagues investigated the effects of 29 different type 1 and type 2 RIPs on polyuridylic acid-directed polyphenylalanine synthesis carried out by purified ribosomes from *Streptomyces lividans*. Only five of them exhibited an IC_50_ below 1 µM including dianthin-32 (331 nM) and as the most effective RIP, crotin-3 (19 nM) from *Croton tiglium* L. seeds while dianthin-30 had an IC_50_ of 6.5 µM [12], indicating that dianthin-30 is more suitable for heterologous expression in bacteria than dianthin-32.

Cenini and colleagues examined the effect of dianthin-32 on ribosomes of the ciliate *Tetrahymena pyriformis* and the amoeba *Acanthamoeba castellanii* [58], and of both dianthin-30 and dianthin-32 on ribosomes of the parasites *Trypanosoma brucei rhodesiense* and *Leishmania infantum* [56]. None of the type 2 RIPs ricin, abrin from *Abrus precatorius* L., modeccin from *Adenia digitata* (Harv.) Engl., viscumin from *Viscum album* L., and volkensin, and the type 1 RIPs gelonin and bryodin had any effect on phenylalanine polymerization by *T. pyriformis* ribosomes while PAP-S (IC_50_ = 1570 nM) and saporin-S6 (IC_50_ = 2630 nM) had a weak effect, momordin a moderate effect (IC_50_ = 300 nM) and dianthin-32 a strong effect (IC_50_ = 30 nM) [58]. The sensitivity of *A. castellanii* ribosomes to these RIPs was higher than that of *T. pyriformis* RIPs but a strong effect was only observed for abrin (IC_50_ = 100 nM), saporin-S6 (IC_50_ = 17 nM) and again, dianthin-32 (IC_50_ = 7 nM), indicating that the efficacy of RIPs on eukaryotic ribosomes is dependent on the RIP and species [58] (Table 2). This was further corroborated by investigating the effect on *T. brucei* and *L. infantum* ribosomes where ricin, modeccin, viscumin, volkensin, gelonin, momordin, mochin from *M. cochinchinensis* (Lour.) Spreng., bryodin, trichokirin and barley (*Hordeum vulgare* L.) RIP had no or only very week effects (IC_50_ > 1.2 µM) while abrin, PAP-S, PAP-R, saporin-S6, saporin-S9 as well as dianthin-30 and dianthin-32 had strong effects, the IC_50_ ranging from 153 nM down to 5 nM [56]. It is notable that different publications describe RIP isolates from *M. cochinchinensis* (Lour.) Spreng. that are called mochin, momochin, momorchin and momorcochin. As sequence data are missing in most of the publications, it remains unclear whether these names describe identical RIPs or isoenzymes. Indeed, in many cases, it is even unclear whether the isolate is a uniform protein. Taylor et al. investigated the rRNA depurination activities of five RIPs using yeast and tobacco leaf ribosomes. PAP-L, dianthin-32, tritin from wheat (*Triticum aestivum* L.) germ, *H. vulgare* L. RIP and ricin A-chain were all active on yeast ribosomes with dianthin-32 being the most active (IC_50_ = 0.019 nM) but only dianthin-32 (IC_50_ = 0.63 nM), PAP (IC_50_ = 0.13 nM) and ricin A chain (IC_50_ = 20.8 nM) were active on tobacco ribosomes [52] (Table 2).

The enzymatic activity of RIPs is not highly specific for 28S rRNA. Other nucleic acids including plasmids, herring sperm DNA (hsDNA), poly(A) and bacterial rRNA might also be recognized as substrate by a number of RIPs [62]. Hartley and co-workers showed that bacterial 23S rRNA can be deadenylated by the type 1 RIPs dianthin-30, dianthin-32, PAP-L and PAP-S but not by the A-chains of the type 2 RIPs ricin and abrin [34]. This was proven by the release of a fragment of 243 nucleotides from the 3′ end of 23S rRNA following aniline treatment of the RNA [34]. It is known that deadenylation renders the surrounding phosphodiester bonds highly susceptible to hydrolysis after treatment with aniline [9,10]. The position of deadenylation by dianthin-32 was found to be A-2660, which lies in a sequence that is highly conserved in all species [34]. Roncuzzi and Gasperi-Campani described a DNA-nuclease activity for dianthin-30, saporin-S6 and gelonin in addition to the *N*-glycosidase activity [63]. In double-stranded, supercoiled pBR322 plasmid DNA, they identified four cleavage sites for dianthin-30 and saporin-S6, and two cleavage sites for gelonin, while ricin did not show any nuclease effect [63]. It is questionable whether the observed DNase activity is indeed present. Instead, the phenomenon might be rather a consequence of an *N*-glycosidase activity, which alters the torsional stress of the supercoiled DNA with subsequent break of the DNA strand [64]. Topologically active dianthin-30 and dianthin-32 was also observed for other plasmids including pGEM4Z and pBlueScript SK^+^ [45,65,66].

The recognition of other substrates can be used to determine the catalytic activity of dianthin directly instead of using indirect effects in translation assays. The principle of all these assays is to determine the amount of adenine released in the presence of the enzyme. Heisler and colleagues developed a colorimetric assay that is conducted in a single multi-reaction incubation step and allows enzyme kinetic measurements [67]. The key step is the conversion of released adenine to adenosine monophosphate by adenine phosphoribosyl transferase. Subsequent reactions finally result in three inorganic phosphate ions per adenine molecule that are quantitated by a color-generating phosphorolysis reaction [67]. The activity for dianthin-30 was determined for the 60S ribosomal subunit, 28S-rRNA, mitochondrial DNA (mtDNA), hsDNA and poly(A). Compared to ricin A-chain and saporin-S3 (which is almost identical to saporin-S6 [21]), the adenine release was highest for dianthin-30 when using mtDNA and hsDNA as substrate, the maximum value reached for hsDNA with 775 picomoles adenine release per picomole of RIP per hour of incubation while ricin A-chain was the most active RIP on 28S-rRNA, exhibiting a release of 185 pmol/pmol/h [67] (Table 3). In this article, the enzymatic activity of a RIP is always expressed as picomoles adenine release per picomole of RIP per hour of incubation (pmol/pmol/h). If other units were used in the literature sources, magnitude and unit were converted accordingly. For more clarity of the unit, we did not reduce the unit fraction to h^–1^, which would be correct but more indistinct. For all three RIPs, the release was below the detection limit of 10 pmol/pmol/h when using the 60S subunit or poly(A) as substrate. Fermani and co-workers quantified released adenine by HPLC/MS ESI [43]. Using this sensitive method, they measured a release of 0.39 to 0.72 pmol/pmol/h for dianthin-30, lychnin, momordin I, ricin A-chain, bouganin, and PAP from rat ribosomes while saporin-S6 was most active exhibiting a release of 1.91 pmol/pmol/h. Adenine release from hsDNA was an order of magnitude higher for dianthin-30, saporin-S6, bouganin and PAP compared to lychnin, momordin-I and ricin A-chain [43], corroborating the results from Heisler and colleagues. Adenine release from poly(A) was only detectable for dianthin-30 (0.54 pmol/pmol/h) and saporin-S6 (>30 pmol/pmol/h) [43]. Weng successfully applied a poly(dA) 30mer and detected released adenine by thin-layer chromatography (TLC) and TLC-densitometry measuring UV absorbance at 260 nm. For the optimization of the assay, deoxy-adenine oligonucleotides with different lengths were used [68]. The release from the poly(dA) 30mer was substantially better than from an RNA that mimics the ribosomal sarcin loop and determined to 110 pmol/pmol/h for dianthin-30 [68]. It is unclear why the strikingly low activity of dianthin-30 observed by Fermani et al. and Heisler et al. for poly(A) [43,67] compared to Weng is attributed to the defined length of 30 residues or to the deoxynucleotides used by Weng. In another study where hsDNA was used as substrate, adenine release by saporin-S3 was 10-fold higher than observed by Fermani et al. for saporin-S6, and 5-fold higher for ricin A-chain [69].

Lubelli et al. described an immuno-polymerase chain reaction assay to detect dianthin and ricin, a method suitable to quantify low amounts of these RIPs (down to 0.01 pg/mL) independent of their enzymatic activity [70]. In this assay, dianthin was detected with a primary and secondary biotin-labelled antibody, and a biotinylated reporter DNA was bound to the secondary antibody using streptavidin as a bridge. Quantitation occurred by polymerase chain reaction of the reporter [70].

As early as 1925, Duggar described the anti-viral effect of pokeweed juice on tobacco plants affected with the tobacco mosaic virus [75]. The active protein was isolated in 1969 [76] and the effect on the larger ribosomal subunit was shown in 1973 [77]. The abbreviation PAP originally stood for *P. americana* L. peptide but was later used as pokeweed antiviral protein. Stirpe et al. first described the antiviral effect of dianthins [1]. The authors infected tobacco leaves with the tobacco-mosaic virus in the presence and absence of dianthin-30 and dianthin-32 and observed almost complete inhibition of virus-mediated lesions at 300 nM [1] (Table 4). This was confirmed by Taylor and colleagues who observed that PAP and dianthin-32 fully inhibit the formation of local lesions at 300 and 3000 nM and more than 80% at 30 nM and 60 nM, whereas tritin, *H. vulgare* L. RIP and ricin A-chain were essentially ineffective [52]. Foà-Tomasi et al. infected HEp-2 cells with herpes simplex virus-1 (HSV-1) or with poliovirus-I in the presence of dianthin-32, PAP-S, gelonin, and a RIP from the seeds of *M. charantia*. All proteins investigated reduced the viral yield, decreased HSV-1 plaque-forming efficiency (Table 4), and inhibited protein synthesis more in infected than in uninfected cells, presumably caused by entering infected cells more easily [78]. As expected, the potency of RIPs on protein synthesis in cells is not the same as in cell-free systems. In the latter, PAP-S, *M. charantia* inhibitor, dianthin-32 and gelonin act in a decreasing order of efficacy whereas in both uninfected and virus-infected HEp-2 cells, dianthin-32 and PAP-S are more potent than the *M. charantia* inhibitor and gelonin, demonstrating that other factors are involved in cells, such as the rate of RIP penetration and degradation [78]. This is undergirded by the observation that binding and uptake of saporin-S6 and momordin by choriocarcinoma BeWo and cervical carcinoma HeLa cells are not correlated to cell toxicity [79]. Batelli and co-workers determined the IC_50_ for inhibition of cell protein synthesis by dianthin-32, saporin-S6, bryodin-R, momordin, gelonin and PAP-S on different cell lines and showed that human trophoblasts and BeWo cells are most sensitive while human embryonal fibroblasts, choriocarcinoma JAR cells and ovarian carcinoma TG cells were less affected. In particular, on fibroblasts, the efficacy of dianthin-32 and saporin-S6 was better than that of the other tested RIPs [79].

A strategy how plants can defend themselves against viruses was experimentally demonstrated by Hong et al. by applying the dianthin-30 coding sequence including the *N*-terminal 23 amino acid signal peptide to engineer resistance to the African cassava mosaic virus in the transgenic tobacco species *Nicotiana benthamiana* Domin by using a promoter that is transactivated by a viral gene product [80]. When challenged with the virus, transgenic plants produced atypical necrotic lesions on inoculated leaves, indicating dianthin-30 expression, moreover, viral DNA accumulation was significantly reduced, and plants exhibit attenuated systemic symptoms from which they recover [80]. By using a potato virus X vector to express the transactivator protein from the African cassava mosaic virus directly in plants, the authors confirmed that amplification of dianthin-30 activity in transgenic plants is indeed mediated by the viral gene product [81]. When dianthin-30 is constitutively expressed in *Nicotiana tabacum* L. cv. Wisconsin 38, the plants are not able to survive, however, dianthin-30 does not hamper the development of rice (*Oryza sativa* L. subsp. *indica* cv. Pusa Basmati1) although all transgenic rice plants harbored and expressed the complete dianthin-30 gene [82]. Notably, the transgenic lines showed reduction of sheath blight symptoms in the range of 29 to 42% [82].

## 3. Dianthin Conjugates and Fusion Proteins

In the last decades, new targeted tumor therapies were developed in the battle against cancer. Such therapies block the growth of cancer cells by interfering with specific pathways required for carcinogenesis and tumor growth. Some procedures are targeted at certain enzymes, proteins, or other molecules involved in the growth and spread of cancer cells and others support the immune system to kill cancer cells or deliver toxic substances to cancer cells. The latter is characterized by a target specific ligand, such as an antibody, growth factor or lectin, and a toxic moiety including radioisotopes, small molecule drugs or protein toxins. Both parts can be conjugated chemically or in the case of proteins, genetically fused [83]. Targeted toxins that contain an antibody or fragment thereof as a ligand are also called immunotoxins.

As type 2 RIPs in addition to the catalytic domain possess a cell binding domain, a number of these proteins are highly cytotoxic for regular cells, in particular, ricin. In contrast, type 1 RIPs such as dianthin, saporin, gelonin, or agrostin from *Agrostemma githago* L. are better suited for the design of targeted toxins since these toxins possess relatively low cytotoxic potential but can be coupled to a targeting moiety to mediate cancer cell binding, internalization and subsequent cell death [17]. Indeed, the median lethal dose in mice after intraperitoneal injection was relatively high for dianthin-30 (12–16 mg/kg) and dianthin-32 (42–46 mg/kg) [46] compared to ricin (0.0024–0.036 mg/kg) [84].

Bolognesi et al. compared anti-lymphocyte immunotoxins containing different RIPs [57]. Immunotoxins were prepared by chemical cross-linking to the F(ab’)_2_ fragment of sheep anti-mouse IgG, their inhibitory effect measured on cell free protein synthesis, and the cytotoxic activity tested on human lymphocytes pretreated with an anti-cluster of differentiation (CD)3 murine monoclonal antibody [57]. Protein synthesis was inhibited by dianthin-32, saporin, PAP-S, gelonin, bryodin, momorcochin, momordin, trichokirin, and ricin A-chain with IC_50_ values between 0.033 nM (saporin) and 0.40 nM (gelonin), dianthin-32 showing an IC_50_ of 0.12 nM. As expected, the corresponding immunotoxins exhibited reduced activity with IC_50_ values between 0.086 nM (saporin-F(ab’)_2_) and 6.2 nM (gelonin-F(ab’)_2_) with the lowest reduction for ricin A-chain-F(ab’)_2_ (1.2-fold) and the highest reduction for gelonin-F(ab’)_2_ (15.5-fold) [57] (Table 2). The activity for dianthin-32-F(ab’)_2_ was reduced 1.4-fold to IC_50_ = 0.167 nM. The immunotoxins inhibited DNA synthesis in phytohemagglutinin-stimulated lymphocytes with IC_50_ values ranging from 0.0026 nM (PAP-S-F(ab’)_2_) to 10 nM (gelonin-F(ab’)_2_), demonstrating that the efficacy on cell free protein syntheses does not correlate with the activity on cells (as PAP-S-F(ab’)_2_ was only the seventh best in blocking protein synthesis) [57] (Table 5). Dianthin-32-F(ab’)_2_ was, after PAP-S-F(ab’)_2_ and saporin-F(ab’)_2_, the third most effective immunotoxin (IC_50_ = 0.011 nM) [57]. In a second study, the authors tested anti-CD30 immunotoxins consisting of a monoclonal antibody chemically coupled to native and recombinant dianthin-30 [54]. Native and recombinant dianthin-30 inhibited cell-free protein synthesis with IC_50_ values of 0.133 nM and 0.227 nM, respectively. For the corresponding immunotoxins, the IC_50_ only increased for recombinant dianthin-30-anti-CD30 (1.054 nM) [54]. Neither native nor recombinant dianthin-30 showed a toxic effect at concentrations up to 10 nM on the CD30 positive cell lines D430B (lymphoblastoid), L428, L540 (both from Hodgkin’s lymphoma) and K562 (myelogenous leukemia). In contrast, both immunotoxins selectively inhibited protein synthesis with IC_50_ values ranging from 0.324 nM to 0.479 nM for native dianthin-30-anti-CD30 and from 0.045 nM to 0.182 nM for recombinant dianthin-30-anti-CD30 on D430B, L428 and L540 cells while K562 cells were not affected more than they were by free dianthin-30 [54] (Table 5). The results demonstrated that dianthin-based targeted toxins can be effectively applied in cell culture. IC_50_ values obtained in cell free assays are not a reliable predictor for cell-based assays. The effect on cells can vary independently of the target receptor expression as other conditions including endocytosis rate, recycling efficiency, degradation rate, endosomal release and toxin sensitivity might also play a role.

Barbieri and colleagues corroborated the diversity of affecting parameters on the efficacy of RIPs [72]. They investigated immunotoxins comprising an anti-CD30 antibody and dianthin-30 (native and recombinant), gelonin, momordin, PAP-S, PD-S2 from *P. dioica* L. leaves, saporin-L1, saporin-S6 or ricin A-chain with regard to enzymatic activity (release of adenine from hsDNA, Table 3), inhibition of cell-free translation and cytotoxicity on L540 cells, and in particular, investigated the effect of the disulfide bonds arising from chemical conjugation. The authors showed that all immunotoxins act on hsDNA and that the activity on hsDNA is only slightly affected by disulfide linkage to the antibody as reduction of these bonds did not substantially increase the activity (only 1- to 6-fold) while inhibition of cell-free protein translation is strongly affected (10- to 63-fold) [72]. The authors further observed that the specific cytotoxicity of the immunotoxins does not correlate with substrate specificity. Among the examined immunotoxins, recombinant and native dianthin-30 were the most effective on L540 cells [72]. In another study, Gilabert-Oriol et al. determined the IC_50_ value of recombinant His-tagged dianthin-30 chemically coupled to a monoclonal anti-calcitonin receptor antibody to 10–20 nM on high-grade glioma SB2b cells. A conjugate of the same antibody with the small molecule toxin monomethyl auristatin E revealed a similar cytotoxicity (IC_50_ = 25.1 nM) [85] (Table 5).

In the case that both ligand and toxin are proteins, heterologous expression of a genetically engineered fusion protein is also an option to produce targeted toxins. Compared to chemically conjugated toxins, the advantage of such an approach is the uniformity of the product, however, expression must be optimized from scratch for each new targeted toxin. Gilabert-Oriol and co-workers expressed two targeted toxins as fusion proteins under identical expression conditions in *E. coli*, however, the yield of a His-tagged dianthin-30 fusion protein with epidermal growth factor (EGF) was nearly two-times higher than the yield for an analogous protein containing saporin-S3 instead of dianthin-30 [87]. Nevertheless, their biological specific activity, monitored in real time by impedance measurement in a cell culture assay on human EGF receptor expressing HER14 cells, was almost equal. A structural comparison revealed five major differences, one resulting in a loop (saporin-EGF) to β-strand (dianthin-EGF) conversion and another introducing a gap in saporin-EGF (after position 57) [87]. Codon usage and toxicity to bacteria were excluded as a cause for different protein expression levels, i.e., minor structural differences might be responsible for the protection of dianthin-EGF from bacterial proteases and therefore may serve as a lead to modify certain domains in type 1 RIP-based targeted toxins [87]. These results were undergirded by Weng, who expressed the same constructs and determined the enzymatic activity of dianthin-EGF and saporin-S3-EGF in an adenine release assay with 67 and 70 pmol/pmol/h respectively, using a poly(dA) 30mer as substrate [68] (Table 3). In another study, the release from hsDNA was 218 pmol/pmol/h for dianthin-EGF and 240 pmol/pmol/h for saporin-S3-EGF [69].

## 4. Endosomal Escape

In contrast to bacterial toxins such as *Pseudomonas aeruginosa* exotoxin A or plant type 2 RIPs such as ricin, type 1 RIPs do not possess a mechanism for retrograde transport to the endoplasmic reticulum (ER) and subsequent cytosolic entry via exploitation of the ER-associated degradation pathway [88]. Further, type 1 RIPs do not possess an acid-sensitive translocation mechanism that allows escape from acidic vesicles, as shown for diphtheria toxin [88,89]. Although the cytotoxic effect of dianthin proves that it is in principle able to reach the cytosol by unknown mechanisms, the efficiency of this endosomal escape is rather low. Therefore, several ideas were investigated to overcome endosomal entrapment and subsequent lysosomal degradation. One approach is the insertion of a cell penetrating peptide to promote the endosomal escape. It was shown that such peptides can improve the anti-tumor activity of saporin-based targeted toxins and concomitantly reduce side effects in mouse tumor models [90]. Another possibility to facilitate the endosomal escape after clathrin-mediated endocytosis is the combined application of certain glycosylated triterpenoids purified from *Gypsophila paniculata* L. and *S. officinalis* L. [91]. These saponins are able to dramatically augment the inhibition of tumor growth by RIP-based targeted toxins in mice, even after 50-fold reduction of the dose [92].

Lorenzetti et al. produced three fusion proteins consisting of the dianthin-30 gene and DNA fragments encoding for the cell penetrating peptides KFT25 (*N*-terminus of protein G of the vesicular stomatitis virus), pHA2 (*N*-terminus of the HA2 hemagglutinin of influenza virus), and pJVE (synthetic peptide) [55]. The fusion proteins retained almost full enzymatic activity in cell-free assays with IC_50_ values of 0.41 nM (dianthin-30), 0.47 nM (KFT25-dianthin), 0.53 nM (pHA2-dianthin-30), and 0.54 nM (pJVE-dianthin-30) (Table 2). Conjugates made by chemically cross-linking KFT25-dianthin and pJVE-dianthin and human transferrin showed greater cell-killing efficiency on Jurkat cells than conjugates of transferrin with wild-type dianthin, but the effect was rather low, namely 3.8-fold and 1.8-fold, respectively (Table 5) [55].

Weng and colleagues tested the effect of the endosomal escape enhancer saponin SA1641 isolated from *G. paniculata* L. on the efficacy of dianthin-30-EGF and other fusion toxins [69]. Without SA1641, they observed an IC_50_ of 0.45 nM for dianthin-30-EGF and 57 nM for saporin-S3-EGF while in the presence of SA1641, the IC_50_ was tremendously decreased to less than 1 fM corresponding to a more than million-fold enhancement in efficacy. In contrast, ricin A-chain-EGF was only enhanced 16-fold and an enzymatically active *P. aeruginosa* exotoxin fragment fused to EGF was not enhanced at all, indicating that intracellular routing to late endosomes is important for the endosomal escape enhancer effect [69] (Table 5). Gilabert-Oriol et al. investigated the effect of another saponin, named SO1861 and isolated from *S. officinalis* L., on chemical conjugates of dianthin-30 with the antibodies cetuximab, panitumumab and trastuzumab [13]. The three immunotoxins did not cause any cytotoxic effects at concentrations up to 10 nM on target cells, however, in combination with SO1861, strong cytotoxicity was observed in all cases with IC_50_ values of 0.0053 nM for dianthin-30-cetuximab and 0.0015 nM for dianthin-30-panitumumab, both tested on EGF receptor overexpressing colorectal carcinoma HCT-116 cells, and 0.023 nM for dianthin-30-trastuzumab on HER2 overexpressing mammary gland ductal carcinoma BT-474 cells (Table 5) [13]. In another study, SO1861 was combined with chemical conjugates of dianthin-30 and a monoclonal anti-calcitonin receptor antibody and tested on high-grade glioma cell lines SB2b, JK2 and U87MG, resulting in IC_50_ values of 0.01 nM, 0.01 nM and 0.02 nM [85] (Table 5). While a conjugate with gelonin showed identical results on U87MG cells, the efficacy of a conjugate with the small molecule toxin monomethyl auristatin E was substantially lower (IC_50_ = 6.3 nM), indicating that SO1861 is suitable to enhance the endosomal escape of RIPs but not of small molecules [85]. Further applications for endosomal escape enhancers can be opened by transferring the genes instead of the proteins into cells. This has already been successfully shown for the saporin cDNA and might also be an option for dianthin cDNA [93,94].

## 5. Mouse Tumor Models

In order to demonstrate the potential of dianthin for targeted tumor therapies, successful application in mouse tumor models would be valuable. In animals, in addition, other aspects such as distribution in the body, liberation, absorption, metabolization and excretion influence efficacy and kinetics of targeted toxins and endosomal escape enhancers, and other aspects such as immunogenicity come into play. At the moment, there are only few studies available. Endosomal escape enhancers that are injected subcutaneously into the neck are rapidly distributed in the body. Already after 10 min, the highest concentration in the kidneys is reached and all other organs showed the peak concentration after 10 to 30 min [95]. Approximately only 1% of the endosomal escape enhancers were detected in the tumor during 10 to 240 min after injection while the remainder was observed in the urine after 30 to 60 min [95]. The toxicity of endosomal escape enhancers is characterized by a clear threshold. All animals treated with 10 mg/kg SO1861 died on the second day of the experiment, while the majority of mice treated with 0.75–5 mg/kg SO1861 only exhibited mild alterations of the liver (e.g., single cell necrosis) but no damage of spleen and kidneys [96]. With regard to dianthin, no toxic effect was observed in mice after intraperitoneal injection of dianthin-32 at doses up to 30 mg/kg body weight [1]. The median lethal dose was determined to be 42–46 mg/kg for dianthin-32 and 12–16 mg/kg for dianthin-30 [46]. Immunogenicity is also an important aspect when using non-human proteins. Strocchi et al. determined the antibody titers in the sera of rabbits immunized with different RIPs. Saporin-S6 was the most immunogenic toxin and resulted in an 11-fold higher titer than dianthin-32 and 29-fold higher titer than bryodin, which was the RIP with the lowest titer among nine tested [25] (Table 6).

His-tagged dianthin-30 genetically fused to EGF was tested in combination with SO1861 in a nude mouse model with subcutaneous HCT-116 tumors [73]. A tumor volume reduction of 96% and complete regression in three of four cases was observed for mice treated with 0.0175 mg/kg dianthin-30-EGF and 1.5 mg/kg SO1861. Histopathological analyses only showed low-grade toxic alterations of the liver such as cytoplasmic degeneration or single cell necrosis [73]. In both placebo and verum, the lymphatic spleen tissue revealed a follicular hyperplasia group, which can be ascribed to an immune response of residual T cells to tumor formation. Pancreas and lung did not show any morphological alterations [73]. The same combination of dianthin-30-EGF and SO1861 was used for the treatment of pancreatic BxPC-3 cell carcinoma in nude mice [71]. Monotherapy with dianthin-30-EGF in the absence of SO1861 resulted on average in a 52% reduction of the tumor volume and no complete regression was observed (three mice had retarded tumor growth and two had continuous tumor growth). However, in the presence of SO1861, the tumor volume was reduced on average by 97% and four out of five mice showed complete regression [71]. Complete blood count analysis did not show suspicious values with the exception of increased platelet count. At the injection site of SO1861, skin hardening was noticed after two therapy cycles, but no skin lesions were observed at the injection site and at the tumor site after six therapy cycles, indicating recovery [71]. Further animal studies with dianthin as the active pharmaceutic agent are required to corroborate its potential role as a cancer cell-killing protein in targeting tumor therapies.

## 6. Conclusions

Dianthin-30 and dianthin-32 are the best investigated dianthin enzymes with dianthin-30 being the lead in the last decade. Three types of activity measurement can be distinguished, direct detection of the enzymatic activity by quantitation of released adenine from various substrates, determination of protein synthesis inhibition in a cell free system, and quantitation of cell viability in cell culture assays. The activity for different RIPs does typically not correlate between the three methods as different properties of the molecule affect the efficacy. Dianthin is a highly active enzyme but as a type 1 RIP, it does not possess a cell binding domain and therefore exhibits low cytotoxicity. This makes dianthin an attractive candidate for targeted tumor therapies as the toxin can be chemically coupled or genetically fused to a ligand that recognizes tumor-specific receptors. Regrettably, most of the targeted dianthin molecules that have been bound to the target receptor are degraded in lysosomes or recycled back to the cell surface after internalization. Endosomal escape enhancers such as particular glycosylated triterpenoids can substantially enhance the release into the cytosol and thus increase the cytotoxicity for target cells by a thousand- up to more than a million-fold. Systematic cell culture experiments provided evidence that dianthin appears to be suitable for targeted tumor therapies. Dianthin can be expressed with higher yield as saporin and is presumably less immunogenic. First studies in mice with targeted dianthin in the presence of endosomal escape enhancers demonstrated the vast potential of this plant enzyme.

## Figures and Tables

**Table 1:** List of dianthin enzymes and of other ribosome-inactivating proteins (RIPs) that are directly compared to dianthins in this article. Data are obtained from the review composed by Schrot et al. [16] except for saporin-S3 [21], crotin-3 [12] and ricin A-chain [22]. In some cases, RIPs described in this review cannot be unequivocally assigned since the identity of that RIP was not fully clarified in the corresponding publication.

RIP	Type	Plant	Plant Source	Molecular Mass (kDa)
dianthin-29	1	*Dianthus barbatus* L.	leaves	29.0
dianthin-30	1	*Dianthus caryophyllus* L.	leaves	29.5
dianthin-32	1	*Dianthus caryophyllus* L.	leaves	31.7
saporin-S3	1	*Saponaria officinalis* L.	seeds	28.6
saporin-S6	1	*Saponaria officinalis* L.	seeds	28.6
saporin-S9	1	*Saponaria officinalis* L.	seeds	28.5
saporin-R1	1	*Saponaria officinalis* L.	roots	30.2
saporin-R2	1	*Saponaria officinalis* L.	roots	30.9
PAP ^1^	1	*Phytolacca americana* L.	leaves	29–30
PAP-S ^1^	1	*Phytolacca americana* L.	seeds	30
PAP-R ^1^	1	*Phytolacca americana* L.	roots	29.8
PD-S2 ^2^	1	*Phytolacca dioica* L.	seeds	29.6
gelonin	1	*Gelonium multiflorum* A.Juss	seeds	30–31
bryodin	1	*Bryonia dioica* Jacq.	roots	29
momordin	1	*Momordica charantia* L.	seeds	31
momorcochin-S	1	*Momordica cochinchinensis* Spreng.	seeds	30
trichokirin	1	*Trichosanthes kirilowii* Maxim.	seeds	27
tritin	1	*Triticum aestivum* L.	germ	30
crotin-3	1	*Croton tiglium* L.	seeds	n. a. ^3^
lychnin	1	*Lychnis chalcedonica* L.	seeds	26.1
bouganin	1	*Bougainvillea spectabilis* Willd.	leaves	26.2
colocin-1	1	*Citrullus colocynthis* (L.) Schrad.	seeds	26.3
asparin	1	*Asparagus officinalis* L.	seeds	29.8–30.5
barley RIP	1	*Hordeum vulgare* L.	seeds	30
ricin A-chain	2	*Ricinus communis* L.	seeds	32
ricin	2	*Ricinus communis* L.	seeds	62.8
abrin-c	2	*Abrus precatorius* L.	seeds	60.1–62.5
modeccin	2	*Adenia digitata* (Harv.) Engl.	roots	57–63
viscumin	2	*Viscum album* L.	leaves	115–125
volkensin	2	*Adenia volkensii* Harms	roots	62

^1^ PAP, pokeweed antiviral protein; without suffix, the protein is obtained from leaves, suffixes -S and -R refer to seeds and roots as source material, respectively. ^2^ PD-S2, *Phytolacca dioica* L. ribosome-inactivating protein number 2 from seeds. ^3^ n. a., information not available.

**Table 2:** Ribosome-inactivating potential of dianthin-30 and dianthin-32 as well as derivates thereof directly compared to other RIPs in several studies. The experimental conditions partly vary substantially between the different publications so that further method information in the source literature must be taken into account for a more detailed comparison of the various RIPs. The activity was typically quantitated by measuring inhibition of the protein synthesis in translation assays, e.g., by (^14^C)-leucine incorporation or poly (U)-directed polymerization of (^14^C)-phenylalanine.

RIP or RIP Fusion Proteins/Conjugates	Translation Inhibition IC_50_ (nM)	Ribosomal Source	Reference
dianthin-30	0.133	rabbit reticulocyte lysate	[54]
	0.18	rabbit reticulocyte lysate	[21]
	0.227 ^2^	rabbit reticulocyte lysate	[54]
	0.29 ^1,2^	rabbit reticulocyte lysate	[14]
	0.31	rabbit reticulocyte lysate	[1]
	0.37 ^2^	rabbit reticulocyte lysate	[14]
	0.41 ^2^	rabbit reticulocyte lysate	[55]
	0.61	rabbit reticulocyte lysate	[14]
	0.35–0.70	wheat germ extracts	[24]
	110	Leishmania infantum	[56]
	153	*Trypanosoma brucei rhodesiense*	[56]
	6478	*Streptomyces lividans*	[12]
dianthin-30-anti-CD30 ^3^	0.1	rabbit reticulocyte lysate	[54]
	1.054 ^2^	rabbit reticulocyte lysate	[54]
KFT25-dianthin-30 ^4^	0.47 ^2^	rabbit reticulocyte lysate	[55]
pHA2-dianthin-30 ^4^	0.53 ^2^	rabbit reticulocyte lysate	[55]
pJVE-dianthin-30 ^4^	0.54 ^2^	rabbit reticulocyte lysate	[55]
dianthin-32	0.02	*Saccharomyces cerevisiae*	[52]
	0.11	rabbit reticulocyte lysate	[1]
	0.12	rabbit reticulocyte lysate	[15]
	0.12	rabbit reticulocyte lysate	[57]
	0.35–0.70	wheat germ extracts	[24]
	0.63	*Nicotiana tabacum*	[52]
	7	*Acanthamoeba castellanii*	[58]
	27	*Leishmania infantum*	[56]
	30	*Tetrahymena pyriformis*	[58]
	66	*Trypanosoma brucei rhodesiense*	[56]
	331	*Streptomyces lividans*	[12]
	991	*Agrobacterium tumefaciens*	[15]
	>1000	*Escherichia coli*	[15]
dianthin-32-F(ab’)_2_	0.167	rabbit reticulocyte lysate	[57]
saporin-S3	0.01–0.02	rabbit reticulocyte lysate	[21]
saporin-S6	0.01–0.02	rabbit reticulocyte lysate	[21]
	0.033	rabbit reticulocyte lysate	[57]
	17	*Acanthamoeba castellanii*	[58]
	33	*Leishmania infantum*	[56]
	116	*Trypanosoma brucei rhodesiense*	[56]
	2630	*Tetrahymena pyriformis*	[58]
	>7133	*Streptomyces lividans*	[12]
saporin-S6-F(ab’)_2_	0.086	rabbit reticulocyte lysate	[57]
saporin-S9	26	*Trypanosoma brucei rhodesiense*	[56]
	31	*Leishmania infantum*	[56]
saporin-R1	0.86	rabbit reticulocyte lysate	[15]
	927	*Escherichia coli*	[15]
	>1000	*Escherichia coli*	[15]
	>1000	*Agrobacterium tumefaciens*	[15]
saporin-R2	0.47	rabbit reticulocyte lysate	[15]
	423	*Escherichia coli*	[15]
	>1000	*Escherichia coli*	[15]
	>1000	*Agrobacterium tumefaciens*	[15]
PAP	0.14	*Nicotiana tabacum*	[52]
	0.29	*Saccharomyces cerevisiae*	[52]
PAP-S	0.037	rabbit reticulocyte lysate	[57]
	17	*Trypanosoma brucei rhodesiense*	[56]
	21	*Leishmania infantum*	[56]
	1040	*Streptomyces lividans*	[12]
	1230	*Acanthamoeba castellanii*	[58]
	1570	*Tetrahymena pyriformis*	[58]
PAP-S-F(ab’)_2_	0.41	rabbit reticulocyte lysate	[57]
PAP-R	40	*Trypanosoma brucei rhodesiense*	[56]
	53	*Leishmania infantum*	[56]
	2005	*Streptomyces lividans*	[12]
gelonin	0.4	rabbit reticulocyte lysate	[57]
	2590	*Leishmania infantum*	[56]
	3330	*Trypanosoma brucei rhodesiense*	[56]
	>3330	*Tetrahymena pyriformis*	[58]
	>3300	*Acanthamoeba castellanii*	[58]
	>11,000	*Streptomyces lividans*	[12]
gelonin-F(ab’)_2_	6.2	rabbit reticulocyte lysate	[57]
bryodin	0.12	rabbit reticulocyte lysate	[57]
	430	*Acanthamoeba castellanii*	[58]
	3330	*Trypanosoma brucei rhodesiense*	[56]
	>3330	*Tetrahymena pyriformis*	[58]
	>3330	*Leishmania infantum*	[56]
	>10,932	*Streptomyces lividans*	[12]
bryodin-F(ab’)_2_	0.247	rabbit reticulocyte lysate	[57]
momordin	0.06	rabbit reticulocyte lysate	[57]
	0.06	rabbit reticulocyte lysate	[15]
	72	*Streptomyces lividans*	[12]
	190	*Escherichia coli*	[15]
	190	*Agrobacterium tumefaciens*	[15]
	300	*Tetrahymena pyriformis*	[58]
	857	*Escherichia coli*	[15]
	1130	*Acanthamoeba castellanii*	[58]
	3330	*Trypanosoma brucei rhodesiense*	[56]
	>3330	*Leishmania infantum*	[56]
momordin-F(ab’)_2_	0.44	rabbit reticulocyte lysate	[57]
trichokirin	0.087	rabbit reticulocyte lysate	[57]
	1265	*Trypanosoma brucei rhodesiense*	[56]
	1747	*Streptomyces lividans*	[12]
	>3330	*Leishmania infantum*	[56]
trichokirin-F(ab’)_2_	0.29	rabbit reticulocyte lysate	[57]
tritin	22	*Saccharomyces cerevisiae*	[52]
	>2200	*Nicotiana tabacum*	[52]
crotin-3	0.2	rabbit reticulocyte lysate	[15]
	13	*Escherichia coli*	[15]
	19	*Streptomyces lividans*	[12]
	40	*Escherichia coli*	[15]
barley RIP	3	*Saccharomyces cerevisiae*	[52]
	1598	*Trypanosoma brucei rhodesiense*	[56]
	>2200	*Nicotiana tabacum*	[52]
	3330	*Leishmania infantum*	[56]
	4922	*Streptomyces lividans*	[12]
ricin A-chain	0.066	rabbit reticulocyte lysate	[57]
	0.17	*Saccharomyces cerevisiae*	[52]
	0.3	rabbit reticulocyte lysate	[55]
	21	*Nicotiana tabacum*	[52]
	800	wheat germ extracts	[50]
ricin A-chain-F(ab’)_2_	0.078	rabbit reticulocyte lysate	[57]
ricin	1030	*Acanthamoeba castellanii*	[58]
	>1700	*Tetrahymena pyriformis^,^*	[58]
	>1700	*Trypanosoma brucei rhodesiense*	[56]
	>1700	*Leishmania infantum*	[56]
	>3300	*Streptomyces lividans*	[12]
abrin	5	*Leishmania infantum*	[56]
	49	*Trypanosoma brucei rhodesiense*	[56]
	100	*Acanthamoeba castellanii*	[58]
	>1700	*Tetrahymena pyriformis*	[58]
modeccin	1700	*Leishmania infantum*	[56]
	>1700	*Tetrahymena pyriformis^,^*	[58]
	>1700	*Acanthamoeba castellanii^,^*	[58]
	>1700	*Trypanosoma brucei rhodesiense*	[56]
viscumin	1700	*Acanthamoeba castellanii*	[58]
	>1700	*Tetrahymena pyriformis^,^*	[58]
	>1700	*Trypanosoma brucei rhodesiense*	[56]
	>1700	*Leishmania infantum*	[56]
volkensin	830	*Acanthamoeba castellanii*	[58]
	1700	*Leishmania infantum*	[56]
	>1700	*Tetrahymena pyriformis^,^*	[58]
	>1700	*Trypanosoma brucei rhodesiense*	[56]
	>3300	*Streptomyces lividans*	[12]

^1^ Dianthin-30 (Δ255–270). ^2^ Recombinant protein. ^3^ CD, cluster of differentiation. ^4^ KFT25, *N*-terminus of protein G of the vesicular stomatitis virus [59], pHA2, *N*-terminus of the HA2 hemagglutinin of influenza virus [60], pJVE, proper name of a synthetic peptide [61].

**Table 3:** Enzymatic activity of dianthin-30 and dianthin-30 derivates in comparison to other RIPs. As in Table 2, the experimental conditions vary from study to study. Adenine release is indicated as picomoles adenine released from the indicated substrate per picomole of RIP in one hour. Most of the data result from end point measurements so that kinetic effects such as substrate depletion might have affected the final outcome.

RIP or RIP Fusion Proteins/Conjugates	Enzymatic Activity Adenine Release (pmol/pmol/h)	Substrate	Reference
dianthin-30	0.54	poly(A)	[43]
	0.57	rat ribosomes	[43]
	<10 ^1^	60S ribosomal subunit	[67]
	<10 ^1^	poly(A)	[67]
	23.99	herring sperm DNA	[43]
	67 ^1^	herring sperm DNA	[71]
	110 ^1^	(dA)_30_	[68]
	140 ^1^	28S-rRNA	[67]
	330 ^1^	mitochondrial DNA	[67]
	644	herring sperm DNA	[72]
	775 ^1^	herring sperm DNA	[67]
dianthin-30-EGF ^3^	42 ^1^	herring sperm DNA	[71]
	67 ^1^	(dA)_30_	[68]
	116 ^1^	herring sperm DNA	[73]
	218 ^1^	herring sperm DNA	[69]
dianthin-30-anti-CD30 ^4^	247 ^1^	herring sperm DNA	[72]
	346	herring sperm DNA	[72]
saporin-S3	<10 ^1^	60S ribosomal subunit	[67]
	<10 ^1^	poly(A)	[67]
	50 ^1^	mitochondrial DNA	[67]
	125 ^1^	(dA)_30_	[68]
	140 ^1^	28S-rRNA	[67]
	312 ^1^	herring sperm DNA	[69]
	397	herring sperm DNA	[69]
	670 ^1^	herring sperm DNA	[67]
saporin-S3-EGF ^3^	70 ^1^	(dA)_30_	[68]
	80 ^1^	herring sperm DNA	[73]
	240 ^1^	herring sperm DNA	[69]
saporin-S6	1.91	rat ribosomes	[43]
	18.3	herring sperm DNA	[74]
	>30	poly(A)	[43]
	37.61	herring sperm DNA	[43]
	439 ^1^	herring sperm DNA	[72]
saporin-S6-anti-CD30 ^4^	176	herring sperm DNA	[72]
saporin-L1	861	herring sperm DNA	[72]
saporin-L1-anti-CD30 ^4^	429	herring sperm DNA	[72]
saporin-L2	934	herring sperm DNA	[74]
PAP-S	519	herring sperm DNA	[72]
PAP-S-anti-CD22 ^4^	115	herring sperm DNA	[72]
PAP-R	<d. l. ^2^	poly(A)	[43]
	0.51	rat ribosomes	[43]
	50.32	herring sperm DNA	[43]
gelonin	12.9	herring sperm DNA	[74]
	579	herring sperm DNA	[72]
gelonin-anti-CD30 ^4^	130	herring sperm DNA	[72]
momordin	<d. l. ^2^	poly(A)	[43]
	0.39	rat ribosomes	[43]
	0.75	herring sperm DNA	[74]
	2.71	herring sperm DNA	[43]
	18	herring sperm DNA	[72]
momordin-anti-CD30 ^4^	1.8	herring sperm DNA	[72]
lychnin	<d. l. ^2^	poly(A)	[43]
	0.72	rat ribosomes	[43]
	2.95	herring sperm DNA	[43]
bouganin	<d. l. ^2^	poly(A)	[43]
	0.48	rat ribosomes	[43]
	37.77	herring sperm DNA	[43]
ricin A chain	<d. l. ^2^	poly(A)	[43]
	0.62	rat ribosomes	[43]
	4.85	herring sperm DNA	[43]
	<10	60S ribosomal subunit	[67]
	<10	poly(A)	[67]
	22	herring sperm DNA	[69]
	27.6 ^1^	herring sperm DNA	[72]
	43	herring sperm DNA	[67]
	185	28S-rRNA	[67]
ricin A chain-EGF ^3^	17 ^1^	herring sperm DNA	[69]
ricin A chain-anti-CD30 ^4^	7.2 ^1^	herring sperm DNA	[72]
ricin	7.5	herring sperm DNA	[74]
	<10	mitochondrial DNA	[67]
	<10	60S ribosomal subunit	[67]
	12	28S-rRNA	[67]
	70	herring sperm DNA	[67]

^1^ Recombinant RIP. ^2^ Less than detection limit. ^3^ EGF, epidermal growth factor. ^4^ CD, cluster of differentiation.

**Table 4:** Effect of dianthin-30, dianthin-32 and other RIPs on virus-infected cells. Readout was the inhibition of plaque formation caused by infected cells or the inhibition of lesion formation in tobacco leaves (*Nicotiana* spec.).

RIP	Concentration (µg/mL)	Virus ^1^	Target	Inhibition(%)	Reference
dianthin-30	0.5	TMV	*N. glutinosa* L.	63	[1]
	1	TMV	*N. glutinosa* L.	91	[1]
	5	TMV	*N. glutinosa* L.	96	[1]
	10	TMV	*N. glutinosa* L.	99	[1]
dianthin-32	0.01	TMV	*N. tabacum* L.	0	[52]
	0.05	TMV	*N. tabacum* L.	0	[52]
	0.1	TMV	*N. tabacum* L.	57	[52]
	0.5	TMV	*N. tabacum* L.	76	[52]
	0.5	TMV	*N. glutinosa* L.	33	[1]
	1	TMV	*N. tabacum* L.	91	[52]
	1	TMV	*N. glutinosa* L.	84	[1]
	2	TMV	*N. tabacum* L.	91	[52]
	5	TMV	*N. glutinosa* L.	86	[1]
	10	TMV	*N. tabacum* L.	100	[52]
	10	TMV	*N. glutinosa* L.	98	[1]
	100	TMV	*N. tabacum* L.	100	[52]
	100	HSV-1	HEp-2 cells	77	[78]
	200	HSV-1	HEp-2 cells	85	[78]
PAP	0.01	TMV	*N. tabacum* L.	0	[52]
	0.05	TMV	*N. tabacum* L.	0	[52]
	0.1	TMV	*N. tabacum* L.	57	[52]
	0.5	TMV	*N. tabacum* L.	76	[52]
	1	TMV	*N. tabacum* L.	91	[52]
	2	TMV	*N. tabacum* L.	91	[52]
	10	TMV	*N. tabacum* L.	100	[52]
	100	TMV	*N. tabacum* L.	100	[52]
PAP-S	100	HSV-1	HEp-2 cells	81	[78]
	200	HSV-1	HEp-2 cells	90	[78]
gelonin	100	HSV-1	HEp-2 cells	31	[78]
	200	HSV-1	HEp-2 cells	83	[78]
momordin	100	HSV-1	HEp-2 cells	70	[78]
	200	HSV-1	HEp-2 cells	81	[78]
tritin	10	TMV	*N. tabacum* L.	36	[52]
	100	TMV	*N. tabacum* L.	23	[52]
barley RIP	10	TMV	*N. tabacum* L.	33	[52]
	100	TMV	*N. tabacum* L.	16	[52]
ricin A-chain	10	TMV	*N. tabacum* L.	20	[52]
	100	TMV	*N. tabacum* L.	25	[52]

^1^ TMV, tobacco-mosaic virus; HSV-1, herpes simplex virus-1.

**Table 5:** Cytotoxicity of dianthin-30 and dianthin-32 as well as derivates thereof in comparison to other RIPs. Cells were incubated with different concentrations of the RIP. The toxin concentration resulting in half maximal effect (IC_50_) was determined by readouts that differ between the studies.

RIP or RIP Fusion Proteins/Conjugates	Cytotoxicity IC_50_ (nM)	Cells/Cell Line	Reference
dianthin-30	>100	D430B	[54]
	>100 ^1^	D430B	[54]
	~30	L540	[54]
	~100 ^1^	L540	[54]
	>100	L428	[54]
	~100 ^1^	L428	[54]
	712 ^1^	Jurkat	[55]
	203 ^1,2^	Jurkat	[55]
	910	H9	[46]
dianthin-30-EGF ^3^	0.45 ^1^	HER14 ^4^	[69]
	<0.0000001 ^1,2^	HER14 ^4^	[69]
dianthin-30-anti-CD30 ^5^	0.479	D430B	[54]
	0.162 ^1^	D430B	[54]
	0.324	L540	[54]
	0.045 ^1^	L540	[54]
	0.355	L428	[54]
	0.182 ^1^	L428	[54]
	7.6	K562	[54]
	>50 ^1^	K562	[54]
dianthin-30-cetuximab	>10 ^1^	HCT-116	[13]
	0.0053 ^1,2^	HCT-116	[13]
dianthin-30-panitumumab	>10 ^1^	HCT-116	[13]
	0.0015 ^1,2^	HCT-116	[13]
dianthin-30-trastuzumab	>10 ^1^	BT-474	[13]
	0.023 ^1,2^	BT-474	[13]
dianthin-30-mAb2C4	10–20	SB2b	[85]
	0.01 ^2^	SB2b	[85]
	0.01 ^2^	JK2	[85]
	0.02 ^2^	U87MG	[85]
transferrin-dianthin-30	1.5 ^1^	Jurkat	[55]
	0.018 ^1,2^	Jurkat	[55]
KFT25-dianthin-30 ^6^	>1000 ^1^	Jurkat	[55]
	712 ^1,2^	Jurkat	[55]
pHA2-dianthin-30 ^6^	>1000 ^1^	Jurkat	[55]
	610 ^1,2^	Jurkat	[55]
pJVE-dianthin-30 ^6^	814 ^1^	Jurkat	[55]
	183 ^1,2^	Jurkat	[55]
dianthin-32	>3200	H9	[46]
dianthin-32-F(ab’)_2_	0.011	PBMC ^7^	[57]
saporin-S3	611 ^1^	HeLa	[86]
	0.24 ^1,2^	HeLa	[86]
	>1000 ^1^	CaSki	[86]
	0.26 ^1,2^	CaSki	[86]
	53 ^1^	HER14 ^4^	[86]
	0.08 ^1,2^	HER14 ^4^	[86]
saporin-S3-EGF ^3^	53 ^1^	HeLa	[86]
	0.00070 ^1,2^	HeLa	[86]
	5 ^1^	CaSki	[86]
	0.00013 ^1,2^	CaSki	[86]
	2.5 ^1^	HER14 ^4^	[86]
	0.00090 ^1,2^	HER14 ^4^	[86]
	57 ^1^	HER14 ^4^	[69]
	<0.0000001 ^1,2^	HER14 ^4^	[69]
saporin-S6-F(ab’)_2_	0.0055	PBMC ^7^	[57]
PAP-S-F(ab’)_2_	0.0026	PBMC ^7^	[57]
gelonin-mAb2C4	0.02 ^2^	U87MG	[85]
gelonin-F(ab’)_2_	10	PBMC ^7^	[57]
bryodin-F(ab’)_2_	0.0288	PBMC ^7^	[57]
momordin-F(ab’)_2_	0.02	PBMC ^7^	[57]
trichokirin-F(ab’)_2_	0.0323	PBMC ^7^	[57]
ricin A-chain	280	Jurkat	[55]
	140 ^2^	Jurkat	[55]
ricin A-chain-EGF ^3^	>1000 ^1^	HER14 ^4^	[69]
	61 ^1,2^	HER14 ^4^	[69]
ricin A-chain-F(ab’)_2_	5	PBMC ^7^	[57]

^1^ Recombinant RIP. ^2^ In the presence of an endosomal escape enhancer. ^3^ EGF, epidermal growth factor. ^4^ HER14, Swiss mouse embryo NIH-3T3 cells transfected with human EGFR. ^5^ CD, cluster of differentiation. ^6^ KFT25, *N*-terminus of protein G of the vesicular stomatitis virus [59]; pHA2, *N*-terminus of the HA2 hemagglutinin of influenza virus [60]; pJVE, proper name of a synthetic peptide [61]. ^7^ PBMC, peripheral blood mononuclear cells.

**Table 6:** Antibody titer of dianthin-32 and other RIPs in the sera of rabbits immunized with RIPs, according to Strocchi et al. [25].

RIP	Antibody Titer (Arbitrary Units)
saporin-S6	44.0
lychnin	21.0
momordin	15.0
colocin-1	7.0
momorcochin-S	6.50
dianthin-32	4.18
PAP-R	2.83
trichokirin	2.16
bryodin-R	1.50
